# Emotional problems in young children during the SARS-CoV-2-pandemic and child attachment

**DOI:** 10.3389/fped.2023.1191032

**Published:** 2023-07-18

**Authors:** Annabel Zwönitzer, Katharina Rost, Jörg M. Fegert, Ute Ziegenhain, Franziska Köhler-Dauner

**Affiliations:** Department of Child and Adolescent Psychiatry/Psychotherapy, University of Ulm, Ulm, Germany

**Keywords:** emotional problems, preschool children, attachment, SARS-CoV-2, mental health

## Abstract

**Background:**

Restrictions imposed by national governments during SARS-CoV-2-pandemic have impacted the mental health of children around the world. Studies have already proven the importance of secure attachment acting as a protective factor in child development. Therefore, children with secure attachment have a higher chance of developing and using appropriate coping mechanisms.

**Objective:**

The goal of this study was to explore the possible connection between the pandemic, as well as child attachment, and young children's emotional health. The general hypothesis assumes a lower rate of emotional problems among children with secure attachment and a higher rate of emotional problems among children with insecure-disorganized attachment, as well as an increase of these problems during the pandemic.

**Method:**

The analysis included *N* = 129 mothers (*M *= 39.98 years) and their children (*M *= 5.19 years). Via an online survey, which was held at different time points during the pandemic, information on the children's emotional problems was retrieved. The survey used the Strengths and Difficulties Questionnaire (SDQ). Data regarding the quality of attachment was collected via the Strange Situations Test (SST). At this point the participating children were approximately 12 months old.

**Results:**

The calculation of a mixed ANCOVA showed, that attachment quality had a significant influence on children's emotional problems [*F*(2, 121) = 4.01, *p* = .021]. The interaction effect between time and child attachment reached significance [*F*(3.45, 208.42) = 3.58, *p* = .011]. The calculation of an additional mixed ANCOVA showed, that the reported emotional problems of girls were higher than those of boys *F*(1, 118) = 4.56, *p* = .035).

**Conclusions:**

Our study shows that there is an association between attachment security and emotional problems and the impact of the SARS-CoV-2 pandemic on the mental health of preschool children in Germany. The emotional problems of all children increased, especially the disorganized attached children had been reported as emotionally stressed in the first lockdown. The results indicate the need for preventive services (for children and parents) to promote and maintain stress coping skills in order to maintain children's mental health in times of crisis.

## Introduction

The day-to-day-lives especially of young families have been impacted by the immediate effects of the SARS-CoV-2 pandemic. Governments were forced to take various measures such as lockdowns, obligatory quarantine and contact restrictions, not only to protect the general public as well as patients with preexisting conditions, but also to keep the spreading of the disease at a minimum. These interventions altered daily routines for many, notably affecting adolescents and children (1, 2). Especially more vulnerable parents with a history of childhood maltreatment are at a higher risk of not having adequate coping strategies in stressful situations, therefore leading to more endangering parenting behavior (3). Since the beginning of the pandemic in 2020, 13 million youths have been afflicted by these measures, with the risk of manifesting as a critical life event. As well as the temporary suspension of schools, kindergardens and other daycare resources, interactions with friends as well as extra-curricular activities were cut to a minimum (4). The possible adverse effects of the pandemic should be taken into consideration, as various research has previously connected the occurrence of psychological issues of children and young adults being exposed to critical life events (5, 6). Munro et al. (7) argue, that the harming effects of school closures overweigh the prevention of viral transmission.

Various studies have indeed documented a worldwide increase of mental health issues in children and the general youth (2, 8–12). Little attention has been received by preschool children and their situation living through a pandemic and the many quarantine measures in Germany so far. However, it has been shown that perceived maternal stress can have an impact on mental health, especially emotional problems, in kindergarden and elementary school children (13). Therefore, they have to be seen as developmentally vulnerable groups. Thus, it's important to address them and the effects of the pandemic and the resulting restrictions on their mental health (14, 15).

Over the course of the first isolation stage, many children were forced to remain at home, constantly surrounded by family members, possibly resulting in increased stress. The high media coverage and news reports regarding the virus pulled most of the attentional capacity of many adults, leading to a decrease in sensitivity towards their children's emotional state (16). Previous studies have recognized children's ability to adapt their own emotional state towards that observed in their parents (17, 18). This results in an increased risk of children not only becoming hyperaware of the negative emotions adults are displaying in their presence, but also a higher tendency to show adverse emotions and problematic patterns of behavior themselves (19, 20). Recent studies show a significant increase in mental health problems in children and adolescents (21). Among other things, an increase in depressive symptoms, anxiety, anger and fear were uncovered in a review by Panchal and colleagues (22). Additionally, 40% of interviewed youths reported a reduced quality of life (23).

A relevant protective factor for mental health and positive development seems to be the quality of attachment between parents and children. Attachment is the lasting emotional relationship between child and caregiver (24). Attachment patterns are activated especially in the case of uncertainty and fear (25). We distinguish between secure, insecure-avoidant, insecure-ambivalent and disorganized attachment patterns. Secure attachment is associated with the feeling of trust and safety in the relationship between child and caregiver. The caregiver can be described as sensitive to the children's needs. Insecure attachment is associated with lower rates of sensitive behavior and is associated with less trust and feelings of insecurity. Secure and insecure attachment are organized attachment patterns, which means that the child has learned a strategy on how to show or not show their needs/feelings to get/gain proximity/closeness and safety. Disorganized attachment is associated with a caregiver who is not always a source of safety but also of fear (24, 26).

Secure attachment provides a protective factor in early childhood in the context of adverse parental circumstances (27). Likewise, a secure attachment protects against the development of anxiety during high family stress and against the development or expression of aggressive behavior (28). Secure attachment seems to contribute to good emotion regulation skills (29). In contrast, meta-analyses confirm associations between insecure attachment and problematic internalizing behaviors (30, 31). Similarly, disorganized attachment seems to be related to more negative emotions, lower emotion regulation abilities and a higher probability of emotional and behavioral problems (29, 32).

The protective effect of secure attachment is based on a better handling of stressful situations and burdens. Previous studies have found that securely attached children are more capable of emotion regulation within the attachment relationship as well as more appropriate use of emotion regulation for themselves (33). Furthermore, previous studies show, that the key to building adequate coping mechanisms and even increased resilience lies within the quality of the attachment between a child and their parent (34). Child attachment seems to play a crucial role for the child's defense mechanisms because of better socio-emotional skills (29, 35) especially when faced with adverse life events, such as the global pandemic. Highly insecure attachment can be interpreted as pathological for development and is thus considered as a risk factor. Highly insecure or disorganized attachment are some of the few predictors of early childhood for the occurrence of future psychopathology in the population (32, 36).

The aim of this study was to investigate the influence child attachment has on children's emotional problems in the context of the pandemic. It is hypothesized that securely attached children generally have less emotional problems, emotional problems of all children worsen over the pandemic, and insecurely/disorganized attached children suffer more from the pandemic in terms of their emotional problems.

## Methods

### Study design

**Figure 1:**
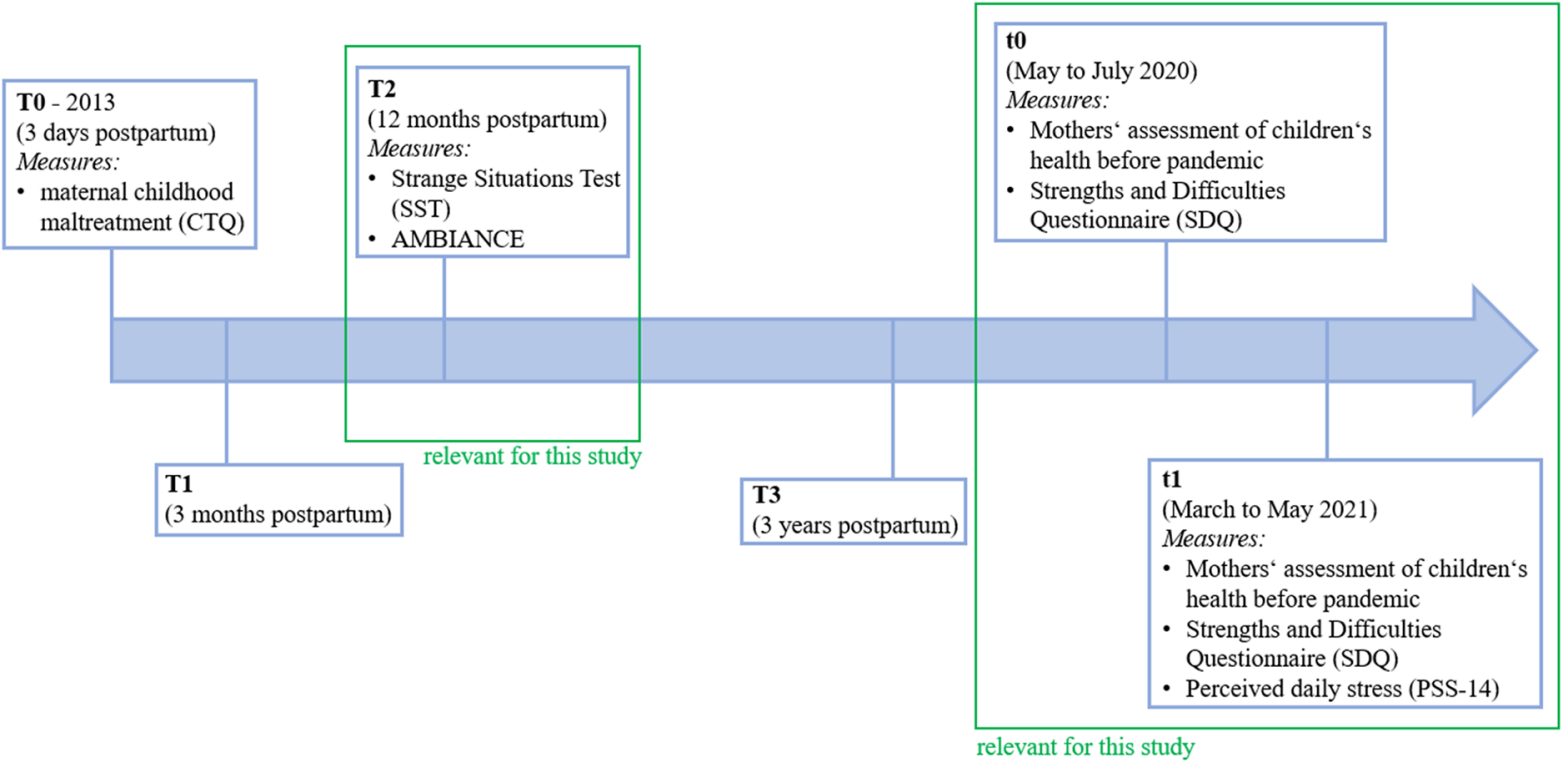
Overview of the study workflow and relevant measurement time points.

The original study aimed at analyzing different risks as well as protective factors concerning the transmission of maternal abuse manifesting in biological, social and psychological factors. [(Blinded)] ensured financing in the time period from October 2013 to March 2017. Requirements and guidelines were adhered by and later approved by the Ethics Committee of [(blinded)] University. The recruiting for the study began in 2013 and was completed at the maternity ward of the [(blinded)] University Hospital. In the following first year, mother-child dyads were screened and monitored. Within the first three days in the hospital the baseline measurement (T0) was carried out retrieving maternal childhood maltreatment experiences through a screening, operationalized by the German version of the Childhood Trauma Questionnaire (CTQ) (37, 38). The following measurements were done after three months (T1), twelve months (T2) and about three years postpartum (T3). At measurement time point T4, home visits were conducted with families and their preschool-aged children. Any increased stress on the families through the SARS-CoV-2-pandemic was evaluated via the “SARS-CoV-2 pandemic survey” from May to July 2020 and March until May 2021, where mothers were also asked to retrospectively assess their children's mental health before the pandemic (Figure 1).

**Figure 2:**
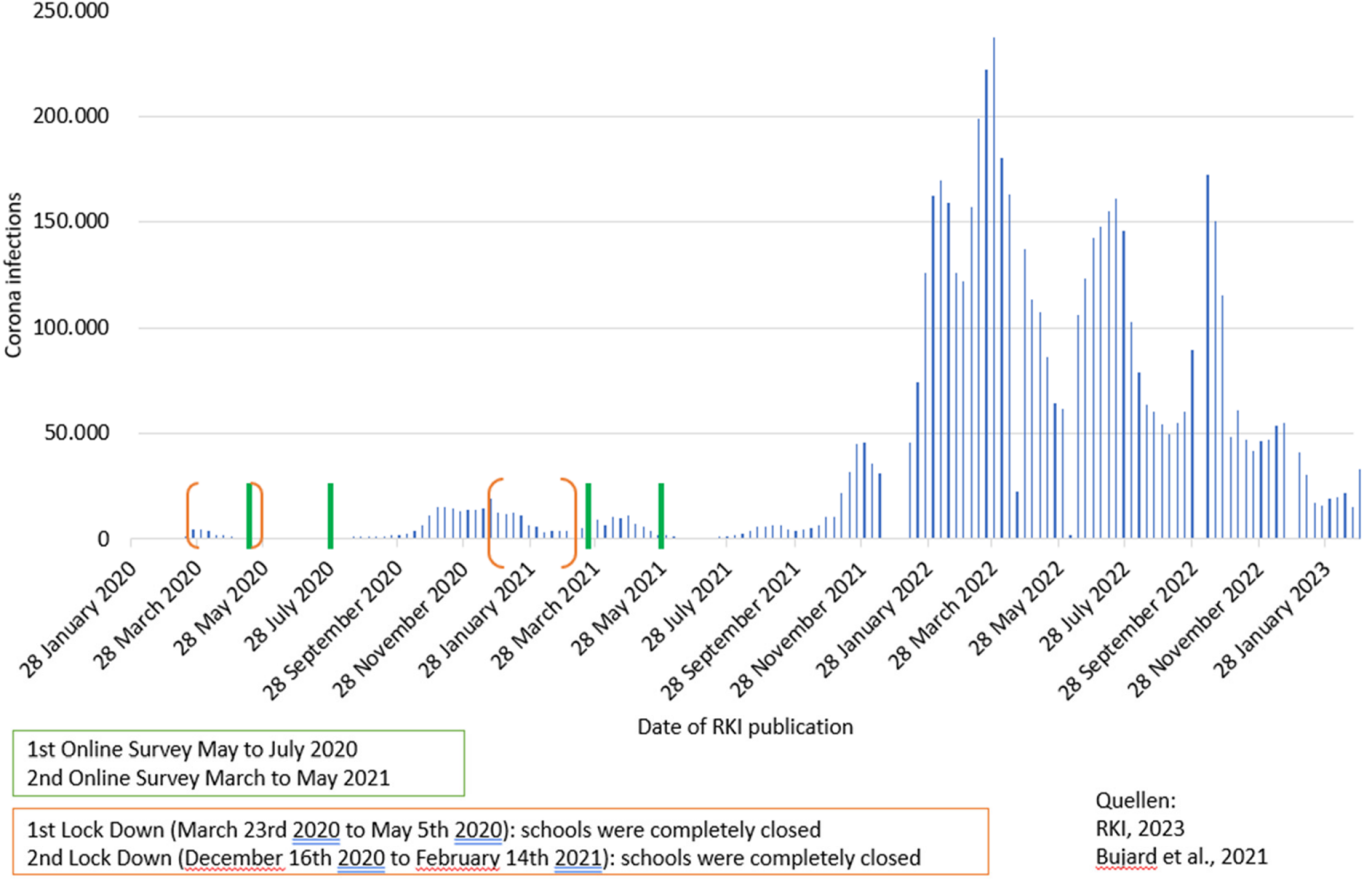
Overview of the survey periods during the pandemic.

The policy measures concerning the SARS-CoV-2 pandemic have been very dynamic. During the first lockdown daycare centers and schools were completely closed from March 23rd 2020 to May 5th 2020, a total of 44 days. This was followed by a partial opening. For most of the children and young people, the time without attendance was between one and a half and three months. After the summer holidays, there were again face-to-face classes. In the second lockdown, which started in early November 2020, day care centers and schools were kept open until mid-December. From December 16th 2020 to February 14th 2021 daycare centers and schools were completely closed. The second lockdown was prolonged several times. Since March 2021 there were differences between the federal states regarding partial opening, especially for primary school children (Figure 2).

The measures relevant to this study were collected at different time points in the original study. Measures of child attachment (SST) and parent-child interaction (AMBIANCE) were conducted, when the children were approximately 12 months old (T2). Children's mental health and parental stress levels were assessed as part of the “SARS-CoV-2 pandemic survey” (Figure 1).

### Participants

*N* = 129 mothers were interviewed in an online survey at two measurement time points. The data originates from a cohort study that began in 2013 with the recruitment of *N* = 533 mothers and their newborns shortly after birth. Mothers over the age of 18, with sufficient skills of the German language and in mental as well as physical health were included. Cases of AIDS, hepatitis, recent alcohol or drug abuse, crucial complications at birth, mental health issues, birthweight lower than 1,500 g and premature birth (less than 37 weeks of pregnancy) were excluded from the study. All mothers gave their written consent for participation. In the fourth survey (T4) the impact of the pandemic on families was assessed with *N *= 158 mother-child dyads. *N *= 129 mothers (carried through and) completed the subsequent “SARS-CoV-2 pandemic survey”, which collected data at two separate time points (t0*: retrospective assessment of the state before the pandemic and current status May 18th–July 31st, 2020, t1*: March 1st–May 31st, 2021) (Figure 1). A few mothers did not complete the survey for reasons such as absence of time or unwillingness to fill out a survey regarding the subject of SARS-CoV-2.

The analysis included *N *= 129 mother-child-dyads. The mothers' average age was *M *= 39.98 years (SD* *= 4.09), with a range from 32 to 50 years. 51% of all mothers had a university degree, while 19% reported a high school diploma, 18% a secondary high school diploma and 12% a lower secondary diploma as their highest certificate of education. The majority of mothers (43%) reported a monthly income of over 4,000 € (<2,000 €: 2%, 2,000–2,500 €: 10%, 2,500–3,000 €: 12%, 3,000–3,500 €: 16%, 3,500–4,000 €: 16%). At t0 the average age of the participating children was *M *= 5.19 years (SD = 0.69) ranging from 4 to 6 years. 48% of the children were female.

## Measures

### Consequences of SARS-CoV-2

The effects on families resulting from the COVID-19 pandemic were collected via the “SARS-CoV-2-pandemic survey”. The questionnaire further assessed various sociodemographic data of the mothers and other close relatives. The mothers were asked to name their age, monthly income, educational level and additional age and gender of other children living in their household. Furthermore, they were asked to name the changes caused by COVID-19 regarding workhours and income, depending on whether the mothers were employed in a sector relevant for the system.

### Strengths and difficulties questionnaire (SDQ)

The German version of the Strengths and Difficulties Questionnaire [SDQ (39);] was used in the “SARS-CoV-2 pandemic survey” at t0 (18 May–31 July 2020) and t1 (1 March–31 May 2021), where mothers were also asked to retrospectively assess their children's mental health before the pandemic. The instrument was used to survey the children's mental health and was completed by their parents. The measurement has an adequate Cronbach's alpha of *α* = .80 (40). The questionnaire measures positive and negative behavior of children using five scales, covering emotional and attention problems, externalizing behavioral problems, as well as difficulties with peers or prosocial behavior. Each scale consists of five items, which are rated on a 3-point Likert scale (0 = not applicable, 1 = partially applicable, 2 = clearly applicable). The total score calculated from these scales, categorizes the child's behavior into either normal, borderline or conspicuous range. This study only included the scale “emotional problems” [Cronbach's alpha *α *= .70 (40);], since only individual items of the other scales of the SDQ were collected in the “SARS-CoV-2-pandemic survey”, in order to minimize the burden on the mothers for answering the questionnaire. The complete emotional problems score was assessed with items such as “frequently complains of headache, stomachache or nausea”, “has a lot of worries, often appears depressed”, “Often unhappy or depressed; often cries”, “nervous or clinging in new situations; easily loses self-confidence” as well as “has many fears; is easily afraid”. When adding up the values, a score resulting between 0 and 3 displayed the normal range, one reaching 4 the borderline range and a range between 5 and 10 categorized the conspicuous range.

### Strange situations test (SST)

The Strange Situations Test was conceptualized by Ainsworth and colleagues (24) to assess children's attachment quality with their mothers. This is assessed by evaluating children's reaction to separation from and reunion with their mother. The Strange Situations Test allows to assess children's behavior towards strangers as the child is left with a stranger for the duration of the separation period. The test can be used for children from 12 to 24 months. In the current study, the Strange Situations Test was performed at T2 when the examined children were approximately 12 months old. The whole procedure was filmed. Through observation, a classification into the different attachment styles (secure attachment, anxious attachment, avoidant attachment and disorganized attachment (24, 26) was executed by a trained coder. Studies appointing trained personnel showed a high inter-rater reliability with agreement rates ranging from 86% to 94% (41–43). There is also evidence for the predictive validity of the Strange Situations Test for developmental outcomes (44).

### Atypical maternal behavior instrument for assessment and classification (AMBIANCE)

The Atypical Maternal Behavior Instrument for Assessment and Classification [AMBIANCE (45);] was used to generate further data on the quality of the maternal interaction with her child. Video recordings of their interaction were made at T2, the child usually at age one. The development of the *AMBIANCE* was based on the Main and Hesse's theory exploring the reasons for frightened, frightening, as well as dissociated parental behavior (46, 47). Physical and emotional disruptions as well as physical withdrawal within the interaction were the foundation of the development of *AMBIANCE*, which is now used as a tool to assess atypical parental behavior, occurring in mother-child interactions (48). *AMBIANCE* is able to analyse disrupted maternal behaviors via code, using a 7-point scale with five dimensions each: (1) affective communication errors, (2) role/boundary confusion, (3) disorganized/disoriented behaviors, (4) negative/intrusive behavior, and (5) withdrawal. Finally, a general score for the level of maternal disruption is created, which is based on intensity as well as frequency displayed in the taped recordings. While Level 4 indicates “non-disrupted”, the levels 5–10 mark the term “disrupted”. All videotaped sessions were analyzed and evaluated by a coder, who had previously been trained by the developers of *AMBIANCE* (45).

### Perceived daily stress (PSS-14)

The mothers' daily stress as perceived by them was assessed via the Perceived Stress Scale 14 (49) at the “SARS-CoV-2 pandemic survey” t1 (March–31 May 2021). The PSS-14 as the original version measures perceived stress in the previous four weeks. It consists of a 5-point-scale from 0 to 4 with a total of 14 items of which 7 are positive and 7 are negative. After the positive items are reversed, a sum score can be calculated from all 14 items. The sum score can range from 0 to 54. In general, high scores are an indicator of high degrees of perceived stress, however, there are no cut-off values as the PSS is not a diagnostic tool. Cohen et al. (49) found a high internal consistency ranging from *α *= 0.84 to *α *= 0.86. This finding is in line with studies which investigated the reliability of the PSS-14 in European samples. In such, an internal consistency ranging from 0.81 to 0.84 was found (50–52). Several studies also show evidence for the convergent, concurrent and criterium validity of the scale (50–52).

## Statistical analyses

The percentage of missing data was 15%. Missing data were replaced by multiple imputation (53). This procedure imputes missing values as a function of all other variables included in the model. Predictive mean matching, logistic regression, and multinomial logistic regression methods were used for variable replacement. 100 sets of imputed data were generated and averaged into a full data set. Multiple imputation was performed using R statistical software (version 4.2.0).

Data was analyzed using SPSS and *p*-values ≤0.05 were considered as significant. Two mixed ANCOVAs were calculated. Children's emotional problems was the dependent variable in both models. Time was the within-subject factor with three measurement time points (before the pandemic vs. t0 vs. t1). Child attachment was the between-subject factor (securely attached vs. insecurely attached vs. disorganized). In the second model, sex was included as an additional independent variable (male vs. female). Mother's age, mother's education, monthly family income, mother-child-interaction (*AMBIANCE global scale*) and mother's stress level (*PSS-14*) were included as control variables in both models. The data was checked for outliers using Cooks distance. No outliers could be examined; therefore, no data was excluded. The assumptions of homogeneity of variances were satisfactory, as assessed by Levene's test (model 1: before pandemic: *p* = .318, t0: *p* = .620, t1: *p* = .275; model 2: before pandemic: *p* = .836, t0: *p* = .067, t1: *p* = .405). Likewise, the assumption of homogeneity of covariances was met, which was tested with the Box-Test (model 1: *p* = .645; model 2: *p* = .500). The Greenhouse–Geisser adjustment was used to correct for violations of sphericity.

## Results

### Descriptive analyzes

The descriptive statistics of all variables are provided in Table 1.

**Table 1:** Descriptive statistics of all variables for *N *= 129 mother-child-dyads.

	*M*	SD	Min.	Max.
Mother's age	39.98	4.09	32	50
Child's age (at t0)	5.19	0.69	4	6
EP before pandemic	1.32	1.39	0	5
EP at t0	2.53	1.84	0	6
EP at t1	2.82	2.04	0	10
**AMBIANCE scales**				
Affective communication errors	3.12	1.35	1	7
Role/boundary confusion	2.05	1.09	1	5
Fearful/disoriented behavior	2.50	1.31	1	6
Intrusive/negative behavior	2.10	1.10	1	5
Withdrawal	3.01	1.33	1	6
Global score	3.69	2.26	1	7
Mother's stress	21.95	9.29	8	48
	***N***	**%**		
**Mother's highest education**				
University degree	66	51		
High school diploma	24	19		
Secondary school diploma	23	18		
Lower secondary school diploma	16	13		
**Monthly family income**				
<2,000 €	3	2		
2,000–2,500 €	13	10		
2,500–3,000 €	16	12		
3,000–3,500 €	21	16		
3,500–4,000 €	21	16		
>4,000 €	55	43		
**Child's sex**				
Female	62	48		
Male	67	52		
**Attachment**				
Secure	76	59		
Insecure	32	25		
Disorganized/disoriented	21	16		

EP, emotional problems.

#### Emotional problems over time and attachment styles

**Figure 3:**
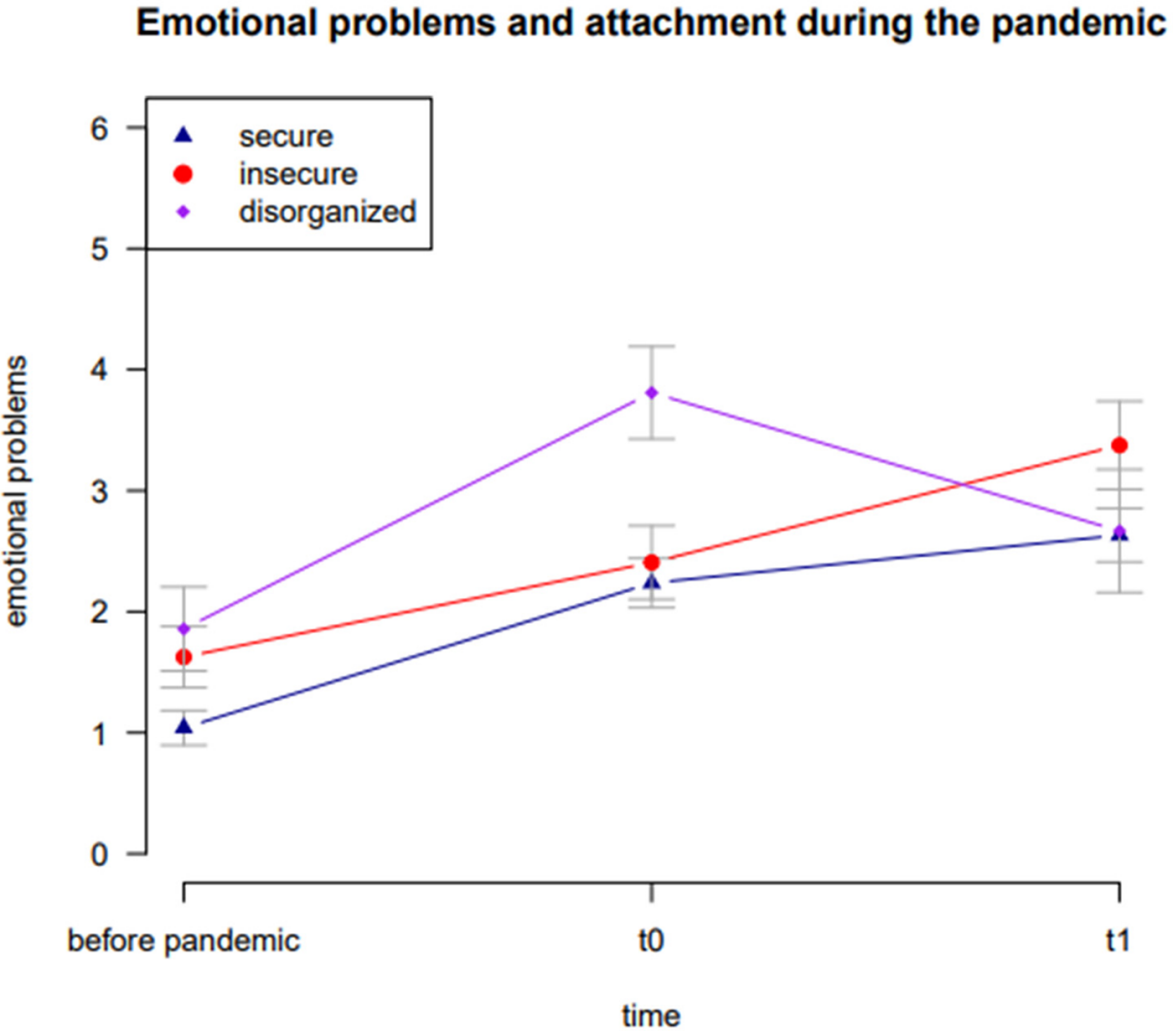
Mean values of children's emotional problems for securely, insecurely and disorganized attached children across the pandemic with standard errors.

Descriptive values of the included variables are shown in Table 2 and illustrated in Figure 3. The results of the mixed ANCOVA are shown in Table 3. The results showed that time did not have a significant impact on the children's emotional problems [*F*(1.72, 208.42) = 2.57, *p* = .087]. As time increased, parents did not report significantly more emotional problems for their children (before pandemic: *M* = 1.32, *SD *= 1.39; t0: *M* = 2.53, SD = 1.83; t1: *M* = 2.82, SD = 2.04). Attachment representation had a significant influence on children's emotional problems [*F*(2, 121) = 4.01, *p* = .021]. The interaction effect between time and attachment reached significance [*F*(3.45, 208.42) = 3.58, *p* = .011].

**Table 2:** Descriptive values of all ANCOVA variables.

		*n*	*M*	SD
Before pandemic	Secure	76	1.04	1.25
	Insecure	32	1.63	1.43
	Disorganized	21	1.86	1.59
t0	Secure	76	2.24	1.78
	Insecure	32	2.41	1.72
	Disorganized	21	3.81	1.75
t1	Secure	76	2.63	1.92
	Insecure	32	3.37	2.06
	Disorganized	21	2.67	2.33

**Table 3:** Results of the mixed ANCOVA with greenhouse-geisser correction.

	*df*	*F*	*p*
Time	1.72, 208.42	2.57	.087
Attachment	2, 121	4.01	.021
Time*Attachment	3.45, 208.42	3.58	.011

Control variables: Mother's age, mother's education, monthly family income, mother-child-interaction and mother's stress level.

#### Sex differences

**Figure 4:**
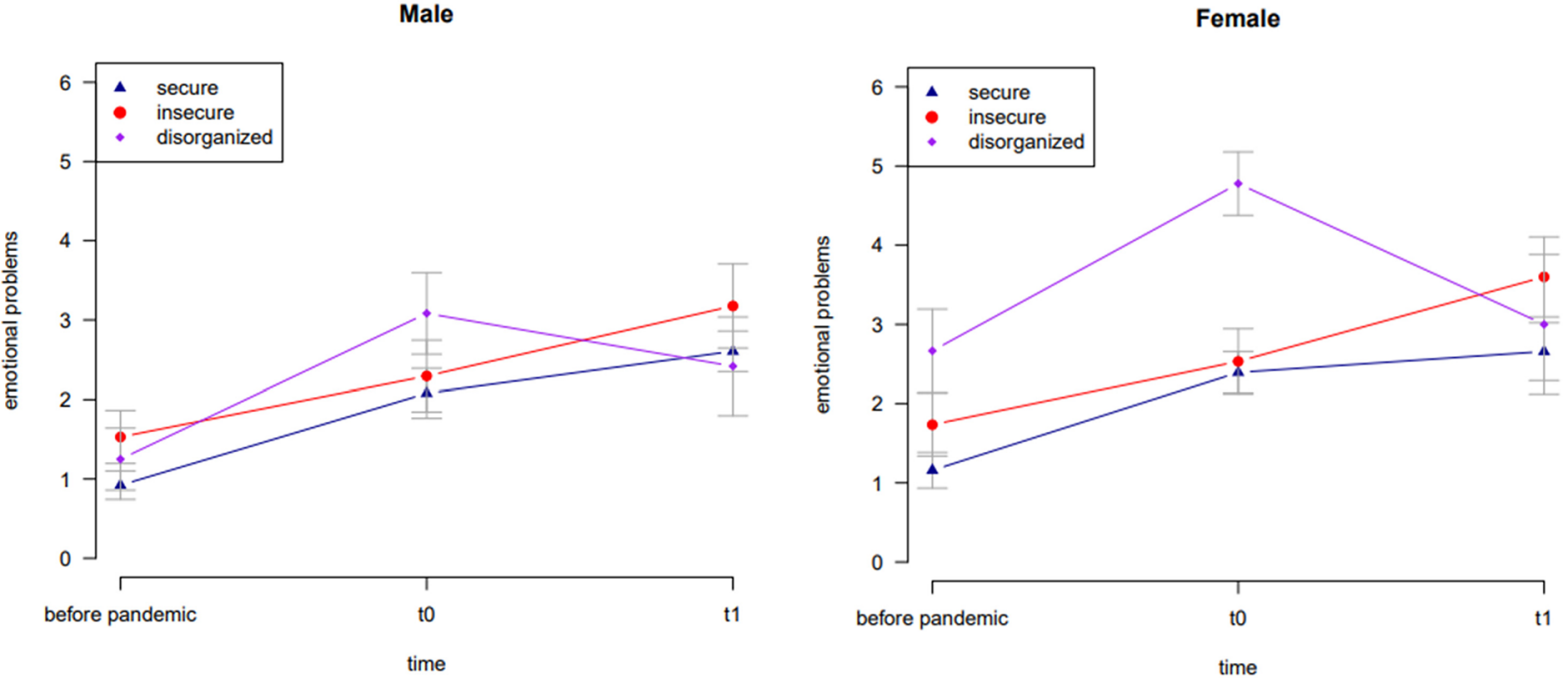
Mean values of males’ and females’ emotional problems for securely, insecurely and disorganized attached children across the pandemic with standard errors.

The results of the second mixed ANCOVA are shown in Table 4 and illustrated in Figure 4. The results showed that time did not have a significant impact on the children's emotional problems [*F*(1.72, 203.39) = 2.55, *p* = .089]. Attachment representation had a significant influence on children's emotional problems [*F*(2, 118) = 4.72, *p* = .011]. Additionally, sex had a significant influence on children's emotional problems [*F*(1, 118) = 4.56, *p* = .035]. The reported emotional problems of girls were higher than those of boys. The interaction effect between time and attachment reached significance [*F*(3.45, 203.39) = 3.69, *p* = .009].

**Table 4:** Results of the mixed ANCOVA with sex as additional independent variable with greenhouse-geisser correction.

	*df*	*F*	*p*
Time	1.72, 203.39	2.55	.089
Attachment	2, 118	4.72	.011
Sex	1, 118	4.56	.034
Time*Attachment	3.45, 203.39	3.69	.009

Control variables: Mother's age, mother's education, monthly family income, mother-child-interaction and mother's stress level.

## Discussion

Especially young families have been impacted by the SARS-CoV-2 pandemic in their daily life since March 2020, leading to diverse challenges (2). Schools, playgrounds and daycare centers had to be closed, contact with family and friends as well as leisure activities had to be further limited (4). There were lots of studies that found increased mental health problems in adolescents and children due to the pandemic (1, 2, 8–12) but there has so far been no study to investigate the influence of attachment on the development of emotional problems during the pandemic.

In line with the results of the studies mentioned above, our investigation has shown a substantial increase in emotional problems during the SARS-CoV-2 pandemic in Germany. The reported emotional problems before the pandemic in our population were lower than the mean emotional problems in the normation population (54). With the duration of the pandemic, we could show an increase of emotional problems. The results are in line with other studies regarding the emotional problems of children (55). Our study shows that there is an overall increase of emotional problems during the SARS-CoV-2 pandemic and an association between attachment security and emotional problems and the impact of the SARS-CoV-2—pandemic on the mental health of preschool children in Germany could be shown. The emotional problems increased significantly more for the disorganized attached children, whereas the emotional problems of the children with secure attachment didn't increase as much. This is interesting because other studies (55) found, that the emotional problems remained on a high level, although the restrictions in terms of the pandemic were getting less restrictive. Therefore, it could be discussed whether the disorganized attached children might be suffering more from the restrictions of being at home and not in the kindergarden, but at the same time recover faster when the kindergarden is open again, being a place symbolizing a second home with hopefully sensitive professionals.

According to literature, secure attachment could be a protective factor for the development of children. In our study, parents of securely attached children reported less emotional problems than the parents of children with (highly) insecure attachments. However, the emotional problems of insecurely attached children did not increase more than those of securely attached children during the pandemic. Regarding our last measurement there has been a decrease of the emotional problems, especially for the disorganized children.

Secure attachment is well-known as a protective factor that buffers developing emotional problems. However, the problems in the context of the SARS-CoV2 pandemic lead to an increase of emotional problems, independent of the assessed attachment style. We could show, however, that insecure attached children are more likely to be in the conspicuous range of emotional problems than securely attached children. There is only one significant finding at our last measurement point, but this could be interpreted as a result of the low numbers in the cells, as we only had a few children meeting criteria for the conspicuous range of the emotional problems scale.

According to other studies on mental health problems of older children during the pandemic (4) there is a significant effect in sex differences: girls suffered more from emotional problems during the pandemic than boys.

Several limitations of the study have to be discussed. First, the study addressed only families who were included in the origin study including families with less risk factors. General conclusions about families as a whole cannot be drawn. Second, the information about emotional problems were conducted through maternal reports due to the young age of the children. Third, there is a lack of information about the mother-child-interaction during the pandemic. The results on the *AMBIANCE* scale and the attachment representation had been assessed when the children were about 12 months old. It would have been interesting to investigate how the actual quality of parent-child-interaction influenced the emotional problems. Thus, the results have to be interpreted with caution.

These limitations notwithstanding, some directions/implications for interventions can be deduced from the study results. The SARS-CoV-2 pandemic had an enhanced impact on young children's emotional problems, highlighting the need for further investment in children's mental health across various sectors, such as kindergardens, preschools and family supportive institutions. In the pandemic children had been used as a shield for other population groups (the elderly and/or those with comorbidities) in order to prevent those vulnerable groups from infection. The result is that there are lots of findings indicating that another vulnerable group has emerged: children with increasing mental health issues.

It is necessary to provide evidence-based interventions especially regarding parenting programs that promote responsive parenting behavior and secure attachment as well. Especially with regards to ongoing problems in the world, like the war in the Ukraine and climate change, there will be more crises developing in the future leading to a general increase in stress on our social systems. According to the German ethics council (2022) there should be an ongoing effort to support children and parents to cope with these circumstances. These adverse childhood experiences that the next generation of children will have to face, will require a functioning social service system with enough resources and preventive working strategies.

## Conclusion

Our study highlights the significant impact of the SARS-CoV-2-pandemic on the mental health of preschool children in Germany. Our findings suggest that pandemic disasters and subsequent containment efforts create a condition that can negatively affect the emotional health of even young children. Because of the increased dependence of children on their parents for stress regulation and the influence of parental stress on children's mental health, special response strategies are needed to address the emotional health needs of young children and their families. Pandemic mitigation measures must take these needs into account. Because pandemic disasters are unique and there are no held-forward interventions for prolonged support and recovery our findings reinforce existing calls [e.g. (4, 9, 56, 57)], to expand preventive services to promote and maintain stress coping skills for both children and their parents in order to maintain children's mental health in times of crisis.

## Data Availability

The raw data supporting the conclusions of this article will be made available by the authors, without undue reservation.
